# Cost-effectiveness analysis of Mucosal Leishmaniasis diagnosis with PCR-based vs parasitological tests in Colombia

**DOI:** 10.1371/journal.pone.0224351

**Published:** 2019-11-04

**Authors:** Liliana Castillo-Rodríguez, Clemencia Ovalle-Bracho, Diana Díaz-Jiménez, Guillermo Sánchez-Vanegas, Sandra Muvdi-Arenas, Carlos Castañeda-Orjuela

**Affiliations:** 1 Observatorio Nacional de Salud, Instituto Nacional de Salud, Bogotá, D.C., Colombia; 2 Hospital Universitario Dermatológico Federico Lleras Acosta, Bogotá, D.C., Colombia; Instituto Butantan, BRAZIL

## Abstract

To estimate the cost-effectiveness of available diagnosis alternatives for Mucosal Leishmaniasis (ML) in Colombian suspected patients. A simulation model of the disease’s natural history was built with a decision tree and Markov models. The model´s parameters were identified by systematic review and validated by expert consensus. A bottom-up cost analysis to estimate the costs of diagnostic strategies and treatment per case was performed by reviewing 48 clinical records of patients diagnosed with ML. The diagnostic strategies compared were as follows: 1) no diagnosis; 2) parasite culture, biopsy, indirect immunofluorescence assay (IFA), and Montenegro skin test (MST) combined ; 3) parasite culture, biopsy, and IFA combined; 4) PCR-miniexon; and 5) PCR-kDNA. Three scenarios were modeled in patients with ML clinical suspicion, according to ML prevalence scenarios: high, medium and low. Adjusted sensitivity and specificity parameters of a combination of diagnostic tests were estimated with a discrete event simulation (DES) model. For each alternative, the costs and health outcomes were estimated. The time horizon was life expectancy, considering the average age at diagnosis of 31 years. Incremental cost-effectiveness ratios (ICERs) were calculated per Disability Life Year (DALY) avoided, and deterministic and probabilistic sensitivity analyses were performed. A threshold of willingness to pay (WTP) of three-time gross domestic product per capita (GDP*pc*) (US$ 15,795) and a discount rate of 3% was considered. The analysis perspective was the third payer (Health System). All costs were reported in American dollars as of 2015. PCR- kDNA was the cost-effective alternative in clinical suspicion levels: low, medium and high with ICERs of US$ 7,909.39, US$ 5,559.33 and US$ 4,458.92 per DALY avoided, respectively. ML diagnostic tests based on PCR are cost-effective strategies, regardless of the level of clinical suspicion. PCR-kDNA was the most cost-effective strategy in the competitive scenario with the parameters included in the present model.

## Introduction

Mucosal Leishmaniasis (ML) is a chronic disease, characterized by lesions that are usually progressive and difficult to diagnose and can lead to irreversible complications [1]. According to the World Health Organization (WHO), an ML case is defined as one that shows clinical signs in the mucosa with parasitological or serological diagnosis; the definitive diagnosis is through visualization of the parasites in biopsy or culture [2]. The sensitivity of these tests varies from 10% to 69% when the methods are combined, mainly due to the presence of a few parasites in the lesions [3]. Molecular tests, such as the polymerase chain reaction (PCR), amplify the Deoxyribonucleic acid (DNA) of the parasite, overcoming the results of classical visualization methods [4–10].

In clinical practice, it is relevant to confirm the ML diagnosis to start treatment with pentavalent antimonials, which is considered the first line of therapy with good results [3,11,12]. Timely treatment could prevent disfiguring lesions, alterations in feeding and obstruction of the airways [3].

There is evidence for the cost-effectiveness of a combination of treatments for visceral Leishmaniasis [13], addressing the use of these treatments through public health policies [14], as well as the cost-effectiveness of preventive strategies in tegumentary Leishmaniasis [15]. However, there are no published studies that evaluate the cost-effectiveness of different diagnostic tests of ML. The objective of the present analysis was to estimate the cost-effectiveness of the available ML diagnostic alternatives in Colombia, including molecular tests, in patients with clinical ML suspicion.

## Methods

Cost-effectiveness analysis of available ML diagnostic alternatives in the country was carried out considering different information sources and estimating the incremental cost per disability-adjusted life year (DALY) avoided. The economic evaluation done, was check under standard guides for reporting health economic evaluations (HEE), with de CHEERS (Consolidated Health Economic Evaluation Reporting Standards) (S1 File).

In Colombia the ML diagnosis is based on clinical and epidemiological criteria (residence and epidemiological link) that allow to establish a diagnostic plan including three test simultaneously: nasal mucosa biopsy, immunofluorescence assay (IFA) with titers greater than or equal to 1:16, and Montenegro skin test (MST) [16]. In Colombia were reported to the national surveillance system 12,000 leishmania cases in 2014 mostly (98%) cutaneous or mucosal [17].

### Parameters

**Fig 1:**
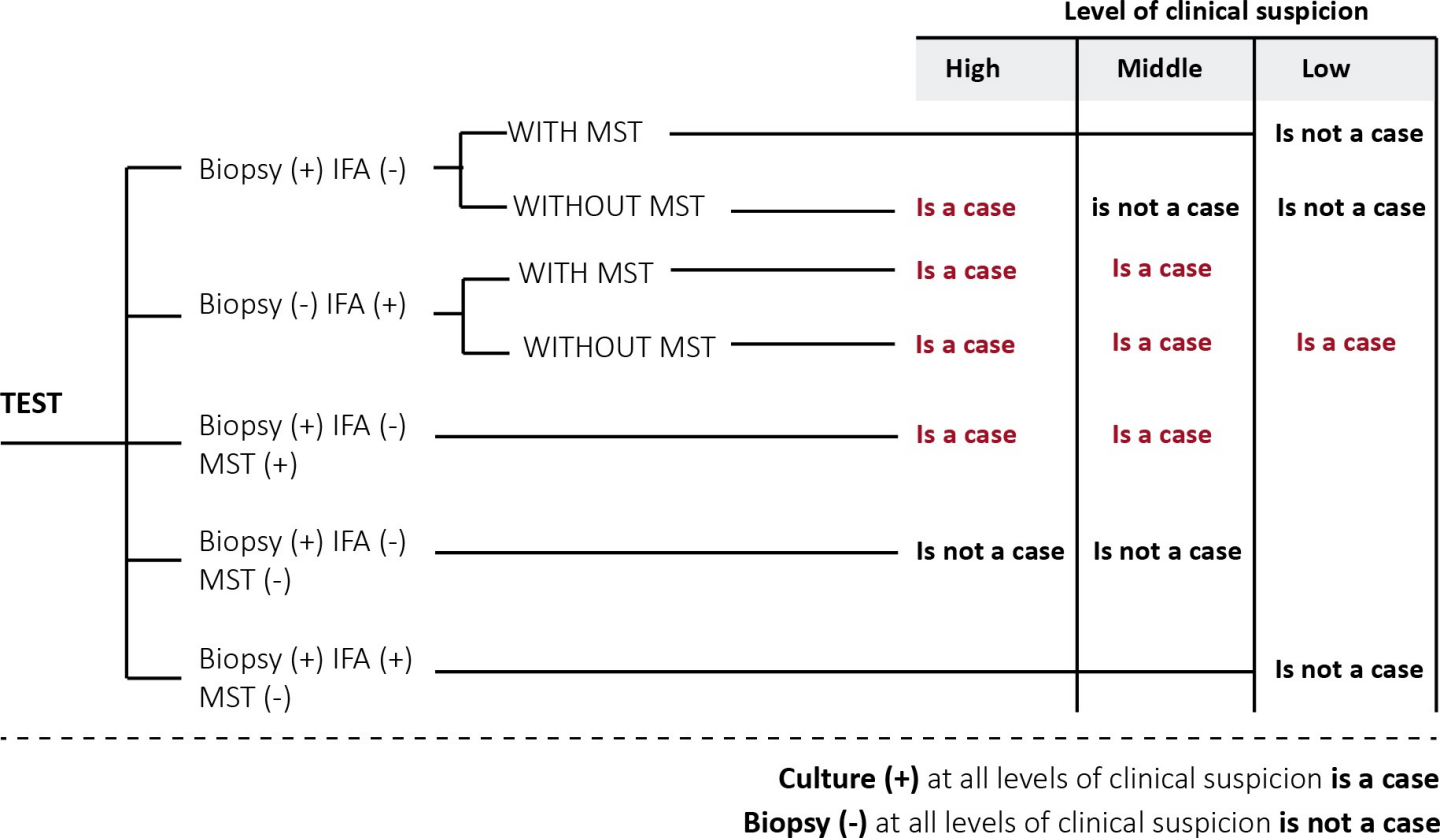
ML case classification according to tests results and level of clinical suspicion.

The economic evaluation model incorporated epidemiological and diagnostic tests’ performance parameters, based on a systematic literature review (Table 1 and S2 File). The parameters were validated in an expert consensus (see below). According to the prevalence of clinical ML suspicion, three scenarios were simulated (high, medium and low ML prevalence). The diagnostic algorithm in each scenario was validated with a group of clinical experts, according to the result of each test: culture, biopsy, IFA and MST (Fig 1). For estimation of the sensitivity and specificity of the diagnostic tests performed simultaneously, a probabilistic discrete event simulation (DES) was programmed to follow-up 10,000 individual patients at each of the levels of clinical suspicion taking into account sensitivity and specificity for each test, identified in the literature. Treatment effectiveness of Glucantime^®^ was reported from a single study in patients with ML in Peru: an open randomized clinical trial was conducted to evaluate the efficacy, safety and tolerance of parenteral aminosidine sulfate AS-(Gabbromicina) 14 mg/kg/day for 21 days compared to intravenous meglumine antimonate MA-(Glucantime^®^) 20 mg/kg/day for 28 days. Cure rates were 8/17 (47%, 95% confidence interval: 23–71%) in the MA group compared to 0/21 in the AS group (P <0.001). It is the only trial that compares two ML treatments including Glucantime^®^, the pentavalent antimony of choice for the treatment in Colombia [18].

**Table 1:** Parameters of the cost-effectiveness model for ML diagnosis in Colombia, 2015.

Parameter	Mean value	Inferior limit	Superior limit	Distribution info^a^	Source
Population with clinical suspicion	200	157	300	Beta (1,2)	[19]
Life expectancy	74	71	77	Beta (2,2)	[20]
**Occurrence of disease**					
Prevalence according to level of clinical suspicion					
High	0.7	0.5	0.8	Beta (2,2)	Expert consensus
Medium	0.3	0.21	0.49	Beta (2,2)	Expert consensus
Low	0.1	0.05	0.2	Beta (2,2)	Expert consensus
Mortality rate due to leishmaniasis	0.0044	0.0039	0.0047	Beta (2,1)	Estimated in the present study
Average age	31				[21]
Incidence	0.00000298	1.87E-01	4.34E-01	Beta (2,2)	[22]
Relapse rate	0.4	0.32	0.48	Beta (2,2)	Expert consensus
**Disability weights of poor health states**					
Mild disfigurement: level 1	0.013	0.006	0.025	Beta (1,2)	Values [23] and expert consensus based on Classification of [24].
Moderate disfigurement: level 2 with pruritus and pain	0.187	0.125	0.264	Beta (2,2)	Values [23] and expert consensus based on Classification of [24].
Severe disfigurement: level 3 with pruritus and pain	0.562	0.394	0.725	Beta (2,2)	Values [23] and expert consensus based on Classification of [24].
**Test**					
**PCR-miniexon**					
Sensitivity	0.867	0.693	0.962	Beta (2,2)	[25]
Specificity	0.967	0.828	0.999	Beta (2,1)	[25]
**PCR-kDNA**					
Sensitivity	0.870	0.72	0.95	Beta (2,1)	Estimated from reference [9,10,26–30]
Specificity	0.940	0.83	0.98	Beta (2,1)	Estimated from reference [9,10,26–30]
**Biopsy**					
Sensitivity	0.217	0.0746	0.437	Beta (1,2)	[9]
Specificity	1	0.478	1	Beta (2,1)	[9]
**Culture**					
Sensitivity	0.10	0.0179	0.4042	Beta (1,2)	[10]
Specificity	1	0.8865	1	Beta (2,1)	[10]
**MST**					
Sensitivity	0.9365	0.7167	0.9889	Beta (2,2)	[10]
Specificity	0.6207	0.44	0.7731	Beta (2,1)	[10]
**IFA**					
Sensitivity	0.83	0.78	0.88	Beta (1,1)	Expert consensus
Specificity	0.75	0.551	0.88	Beta (1,1)	[10]
**Treatment**					
Treatment start	0.85	0.8	1.00	Beta (1,2)	Expert consensus
RR of treating ML patients with Glucantime^®^	0.53	0.338	0.829	Beta (1,2)	Estimated from reference [18]
Effectiveness of treating ML patients with Glucantime^®^	0.47	0.171	0.662	Beta (1,1)	[18]
Abandonment rate	0.30	0.24	0.36	Beta (2,2)	Expert consensus
**Other assumptions**					
Discount rate	0,03				[31,32]
GDP*pc* (US$)	5,265.03				[33]
**Costs of diagnostic alternatives (In US$)**					
**Alternative 1:** Sample collection (Biopsy) + Culture + stains + IFA + MST alt_1	172.40			Gamma (SD: 17.24)	Estimated in the present study
**Alternative 2:** Sample collection (Biopsy) + Culture + stains + IFA alt_2	162.57			Gamma (SD: 16.26)	Estimated in the present study
**Alternative 3:** Sample collection (Biopsy) + stains + IFA alt_3	128.91			Gamma (SD: 12.89)	Estimated in the present study
**Alternative 4:** PCR-miniexon	128.77			Gamma (SD: 12.88)	Estimated in the present study
**Alternative 5:** PCR-kDNA	128.77			Gamma (SD: 12.88)	Estimated in the present study

^a^ Probabilities have beta distributions, while cost assumed gamma distributions. SD: standard deviation)

### Costs estimation

We selected 48 clinical records of patients who met the clinical criteria for ML treated at the *Hospital Universitario Dermatológico Federico Lleras Acosta* (HUDFLLA) during 2001–2014. The clinical criteria included patients with clinical suspicion or suggestive biopsy and those compatible with ML; therapeutic response to treatment; parasitological visualization; cutaneous Leishmaniasis scar; and endemic origin.

The estimated cost of care per patient was based on a bottom-up cost with an ingredient-based approach [32], including the treatment of adverse effects associate to ML medications. We elaborated and piloted an instrument for data collection about the number of consultations, frequency of use, dosage and presentation of medications, clinical and paraclinical examinations, and interconsultations, as well as any procedure or intervention performed.

The price information was provided by the HUDFLLA and the *Seguro Obligatorio de Accidentes de Tránsito* (SOAT) 2015 tariffs [34]. We calculated averages and median costs as summary measures, as well as the standard deviation and interquartile ranges. The price of Glucantime^®^ was reported by the Leishmaniasis program at the National Ministry of Health [35]. Other medicine prices were extracted from the *Sistema de Información de Precios de Medicamentos* (SISMED) [36].

The present analysis had the perspective of the third payer (Colombian Health System), so only direct costs related to the care of the disease were included. All costs were expressed in 2015 US dollars (exchange rate of US $1 = 3,149.47 Colombian pesos [COP]). A discount annual rate of 3% for both costs and results was included in the model according to the recommendations from the international literature [31,32].

### Cost-effectiveness model

**Fig 2:**
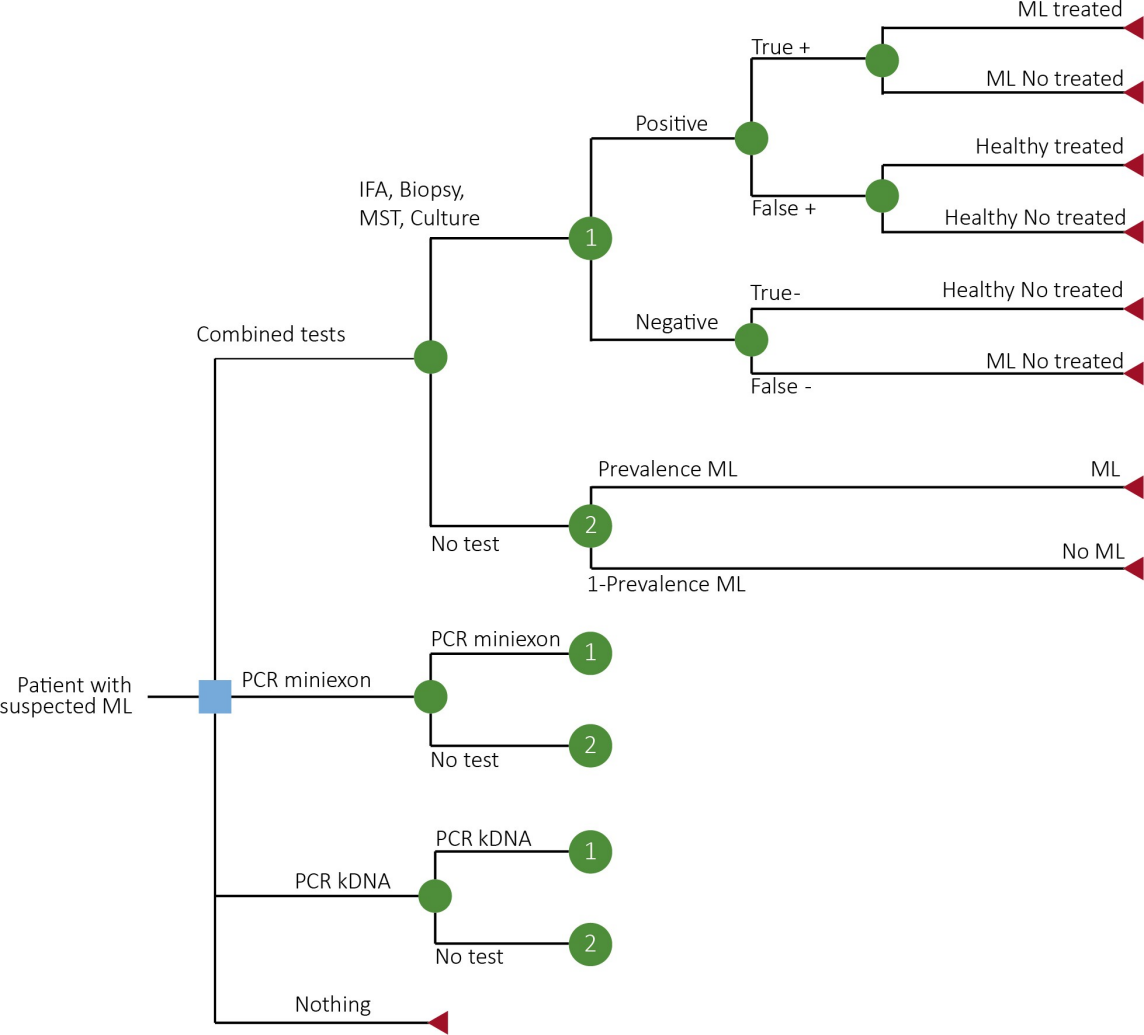
Decision tree for the economic evaluation of ML diagnosis in a cohort of ML suspected patients. Nodes 1 and 2, are repeated for each decision tree arm.

**Fig 3:**
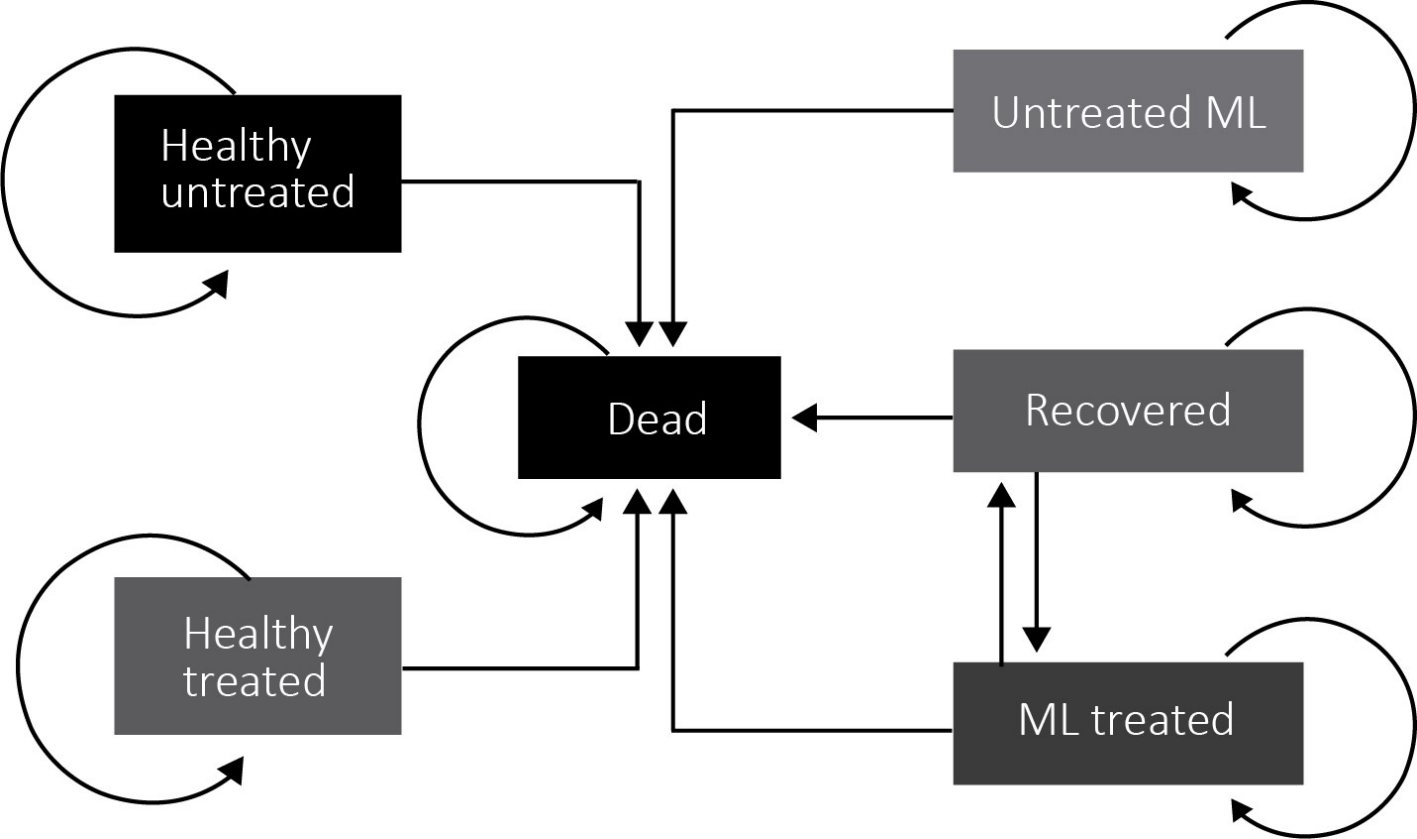
Markov model for the economic evaluation of ML diagnosis in a cohort of ML suspected patients.

A cost-effectiveness model (S3 File) was built combining different modeling strategies that included a decision tree (Fig 2) and a Markov model (Fig 3) in a cohort of ML suspected patients according to the level of clinical suspicion.

#### States considered

The Markov model included annual cycles of transition between six mutually exclusive states: 1) Healthy untreated; 2) Healthy treated; 3) ML treated; 4) ML untreated; 5) Recovered; and 6) Dead. These states were reached after the ML suspected patient has had an adequate or inadequate diagnosis, according to each of the alternatives modeled.

#### Alternatives to compare

Six alternatives were selected: 1) Not diagnosed in ML suspected patients; 2) Parasitological culture, Biopsy, IFA, and MST combined in parallel; 3) Parasitological culture, Biopsy and IFA combined in parallel; 4) Biopsy and IFA combined in parallel; 5) PCR-miniexon; and 6) PCR-kDNA. Diagnostic tests for mucosal leishmaniasis are limited in Latin America, in this study all the tests recommended by the Pan American Health Organization and the World Health Organization were used [37].

#### Time horizon and included health outcomes

An annual model was run up to a life expectancy of 74 years [19] (95% CI 70.95–77.1) to include all the relevant health results. Patients were followed since age 31, which corresponded to the average age of diagnosis reported in the Epidemiological Surveillance System (*Sivigila*)[20]. Health outcomes were assessed as DALYs, which corresponded to the sum of the years of life lost (YLL) and the years of life lived with disability (YLD), obtained by each of the arms and cohorts evaluated, with the half-cycle adjustment to avoid overestimations. YLDs were estimated according with the duration of the disease and levels of disability (disfigurement due to ML), according with the tables from Global Burden of Disease Study (GBD) [23].

### Estimating the Incremental Cost-Effectiveness Ratio (ICER)

We calculated the ICER defined as the ratio between the cost difference and the difference in health outcomes:

$$ICER=\left(\frac{{ANC}_{2}-{ANC}_{1}}{{AE}_{2}-{AE}_{1}}\right)$$

where:

ANC_2_ = average net costs of diagnosing with alternative 2,

ANC_1_ = average net costs of diagnosing with alternative 1,

AE_2_ = average effectiveness of diagnosing with alternative 2,

AE_1_ = average effectiveness of diagnosing with alternative 1.

With six mutually exclusive alternatives, cost-effectiveness was evaluated in a competitive scenario, ordering the alternatives from least to most costly and evaluating incremental cost-effectiveness with respect to the previous non-dominated alternative. As a cost-effectiveness threshold or willingness to pay (WTP), the Colombian gross domestic product per capita (GDP*pc*) value of 2015, which was US $ 5,265.03, as recommended by the WHO macroeconomic commission, was considered three times (US$ 15,795.09) [38].

### Sensitivity analysis

The deterministic sensitivity analysis (DSA) was performed from the comparison between do nothing and PCR-kDNA (tornado diagram) for each of the levels of clinical suspicion. In addition, a scenario analysis was performed by varying the parameter "cost of the untreated case" and analyzing the changes in the ICER.

A probabilistic sensitivity analysis (PSA) for all included parameters was carried out considering its probability distribution, using a Monte Carlo simulation. A total of 25,000 probabilistic models were randomly estimated varying all the parameters. The mean value and a 95% uncertainty range of the results were reported. The sensitivity and specificity parameters of the diagnostic tests were sensitized considering their autocorrelation (the probability of positivity of one test is not independent of the result of another test). Acceptability curves were constructed with the decision rule based on the Net Health Benefit (NHB).

For estimation of the costs of the disease, the average costs per patient were calculated and their respective interquartile ranges were calculated, which were computed via bootstrapping, with 10,000 iterations, to estimate the median costs of the disease per case. The processing and analysis of all the information and the model building were performed in MS Excel^®^ (S2 File).

### Expert consensus

A consensus of clinical experts (dermatologists, bacteriologists and epidemiologists specializing in tropical diseases with emphasis on Leishmaniasis) and economic evaluations (health economists and administrators) were made. The consensus validated parameters where no quality evidence was available or there was lack of evidence, and they validated the structure of the model and the results of the cost-effectiveness analysis.

Agreements in the consensus were valued as follows: if there was agreement less than 60%, the question was reformulated and it was returned for a vote; in this intervention, each expert counted once minute. If a 60% agreement was not reached in the second round, it was considered a lack of agreement. In open-ended questions, three rounds produced an agreement. Observers were allowed to participate in two moments: at the end of the validation of the parameters and the model, or when the experts requested it.

### Ethics statement

This research project was approved by the Research Ethics Committee of the HUDFLLA N° 14 December 10^th^, 2014 (Internal communication DGD-2003-048) based on the Declaration of Helsinki and the current Colombian legislation (Resolution 8430/1993). The study was classified as minimal risk and was carried out from information extracted from secondary sources, including the clinical record of ML cases. All the records were anonymized and coded, and never were identified with personal or contact information. Prior written informed consent for the use of information from clinical histories had been obtained at the time of diagnosis from patients included in this study. Other information sources include administrative public databases such as scientific literature, Sivigila, mortality database from the National Institute of Statistics (DANE), and Central Bank´s variables.

## Results

The average of the median cost of care of a ML patient was estimated in US$ 432.71 (95% CI US$ 376.86–578.93), where the treatment corresponds to 53.72% of the total cost (Table 2). The cost-effectiveness analysis of the base case at a low clinical suspicion level (Table 3) showed that the costliest alternatives included Culture + Biopsy + IFA. Both alternatives (with and without MST) were strong dominated because they were more expensive and less effective than PCR- kDNA. However, Biopsy + IFA and PCR miniexon presented extended dominance (an ICER larger than the next cost-effective strategy) and was excluded from the comparison. The ICER for PCR-kDNA was US$ $7,909.39.

**Table 2:** Total cost estimates by ML cases in Colombia.

Cost Items	Cost (USD $)
Medical consultations	7,741.21
Procedures	40.01
Diagnostic tests (labs, X-rays, Electrocardiogram, Computed axial tomography)	4,728.79
Medicines	14,519.39
Total cost	27,029.40

**Table 3:** Cost-effectiveness results for ML diagnostic tests at low clinical suspicion, Colombia, 2015.

Alternative	Cost (US$)	DALYs	Incremental Cost (US$)	DALYs avoided	ICER (US$)
**Do nothing**	5,294.33	400.78			
**Biopsy + IFA**	36,327.53	398.73			Extended dominated^a^
**PCR-miniexon**	44,580.48	396.15			Extended dominated^a^
**PCR-kDNA**	45,913.10	395.64	40,618.77	5.14	7,909.39
**Culture + biopsy + IFA + MST**	46,511.95	398.24	598.84	- 2.60	Dominated
**Culture + biopsy + IFA**	46,917.55	397.23	405.61	-1.59	Dominated

^a^This value is excluded by extended dominance; the cost-effectiveness of the alternative PCR-kDNA was re-estimated with the previous alternative (do nothing).

At medium and high suspicion levels (Table 4 and Table 5, respectively), the costliest alternatives were PCR-kDNA and PCR-miniexon. In this comparison, the alternatives Biopsy + IFA; Culture + biopsy + IFA; Culture + biopsy + IFA + MST; and PCR-miniexon (Table 3) presented extended dominance because the ICER was larger than the next cost-effective strategy. The cost-effective strategy was PCR-kDNA at a cost of US$ 5,5596.33 and US$ 4,458.92 per avoided DALY, compared to Do nothing.

**Table 4:** Cost-effectiveness results for ML diagnostic tests at medium clinical suspicion, Colombia, 2015.

Alternative	Cost (US$)	DALYs	Incremental Cost (US$)	DALYs avoided	ICER (US$)
**Do nothing**	15,876.84	451.29			
**Biopsy + IFA**	49,837.78	448.47	33,960.94	2.82	Extended dominated^a^
**Culture + biopsy + IFA**	62,691.23	446.36	12,853.45	2.10	Extended dominated^a^
**Culture + biopsy + IFA + MST**	67,595.20	445.19	4,903.97	1.18	Extended dominated^a^
**PCR-miniexon**	73,435.89	441.28	5,840.69	3.90	Extended dominated^a^
**PCR-kDNA**	5,634.42	440.54	2,198.52	0.75	5,5596.33

^a^This value is excluded by extended dominance; the cost-effectiveness of the alternative PCR-kDNA was re-estimated with the previous alternative (do nothing)

**Table 5:** Cost-effectiveness results for ML diagnostic tests at high clinical suspicion, Colombia, 2015.

Alternative	Cost (US$)	DALYs	Incremental Cost (US$)	DALYs avoided	ICER (US$)
**Do nothing**	37,041.86	552.30			
**Biopsy + IFA**	82,560.94	545.82	45,519.08	6.49	Extended dominated^a^
**Culture + biopsy + IFA + MST**	99,452.48	543.50	16,891.53	2.32	Extended dominated^a^
**Culture + biopsy + IFA**	99,599.50	542.61	147.02	0.88	Extended dominated^a^
**PCR-miniexon**	131,096.64	531.55	31,497.14	11.06	Extended dominated^a^
**PCR-kDNA**	135,041.94	530.32	3,945.29	1.23	4,458.92

^a^This value is excluded by extended dominance; the cost-effectiveness of the alternative PCR-kDNA was re-estimated with the previous alternative (do nothing)

**Fig 4:**
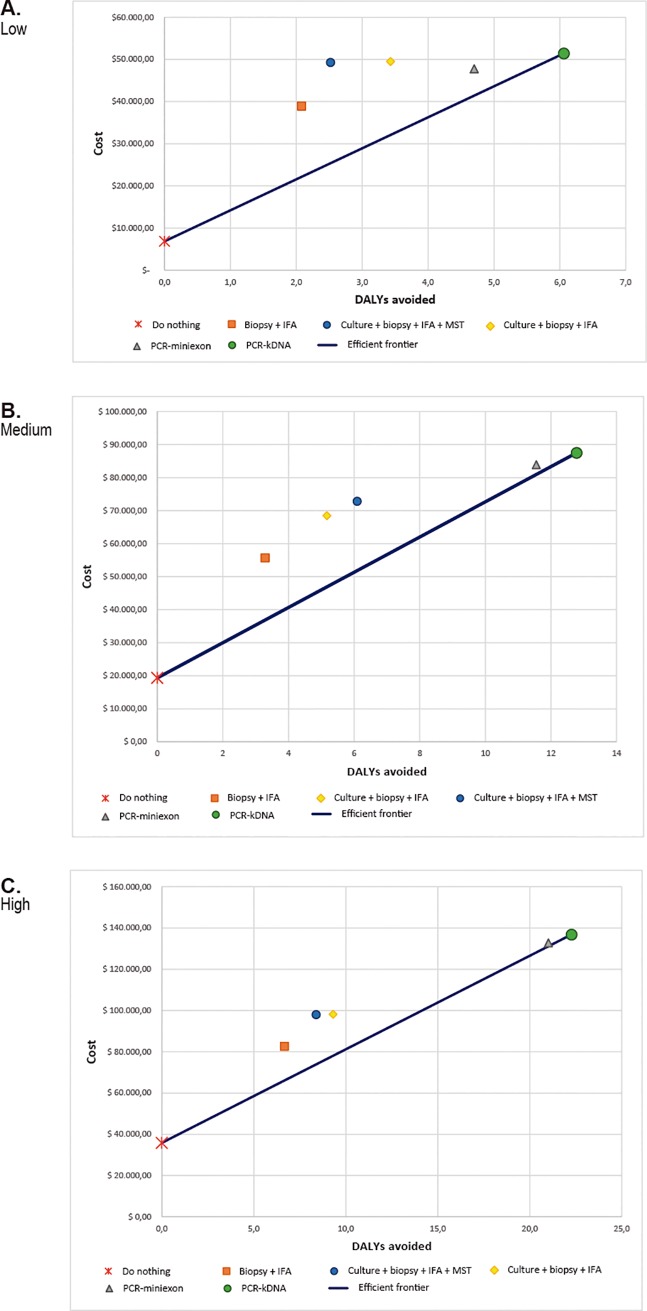
Efficient frontier for ML diagnosis test according to the levels of clinical suspicion, Colombia, 2015. (A) Low, (B) Medium, (C) High.

The efficient frontier analysis (Fig 4) shows how the only alternative within the efficient frontier for all clinical suspicion level was PCR-kDNA with the ICER always in cost-effective area (values less than US$ 15,795 per DALY avoided) comparing with non-diagnosis. All other alternatives, including PCR- miniexon were most of the times extended dominated.

### Deterministic sensitivity analysis (DSA)

**Fig 5:**
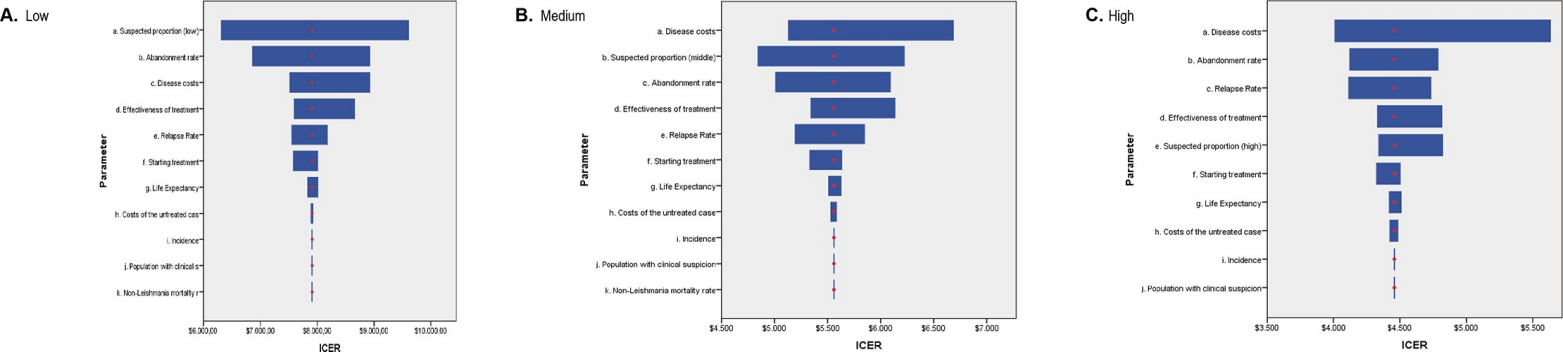
Tornado diagram according to the levels of clinical suspicion, comparing PCR-kDNA versus do nothing, Colombia, 2015. (A) Low, (B) Medium, (C) High.

For all the three levels of clinical suspicion (low, medium and high), the results of the tornado diagram are presented, for the comparison PCR-kDNA versus do nothing (Fig 5). The disease cost per patient is the parameter that most influences the results at a high and medium level of clinical suspicion, while at low suspicion level, the results are more sensitive to proportion of suspected patients.

### Probabilistic sensitivity analysis (PSA)

**Fig 6:**
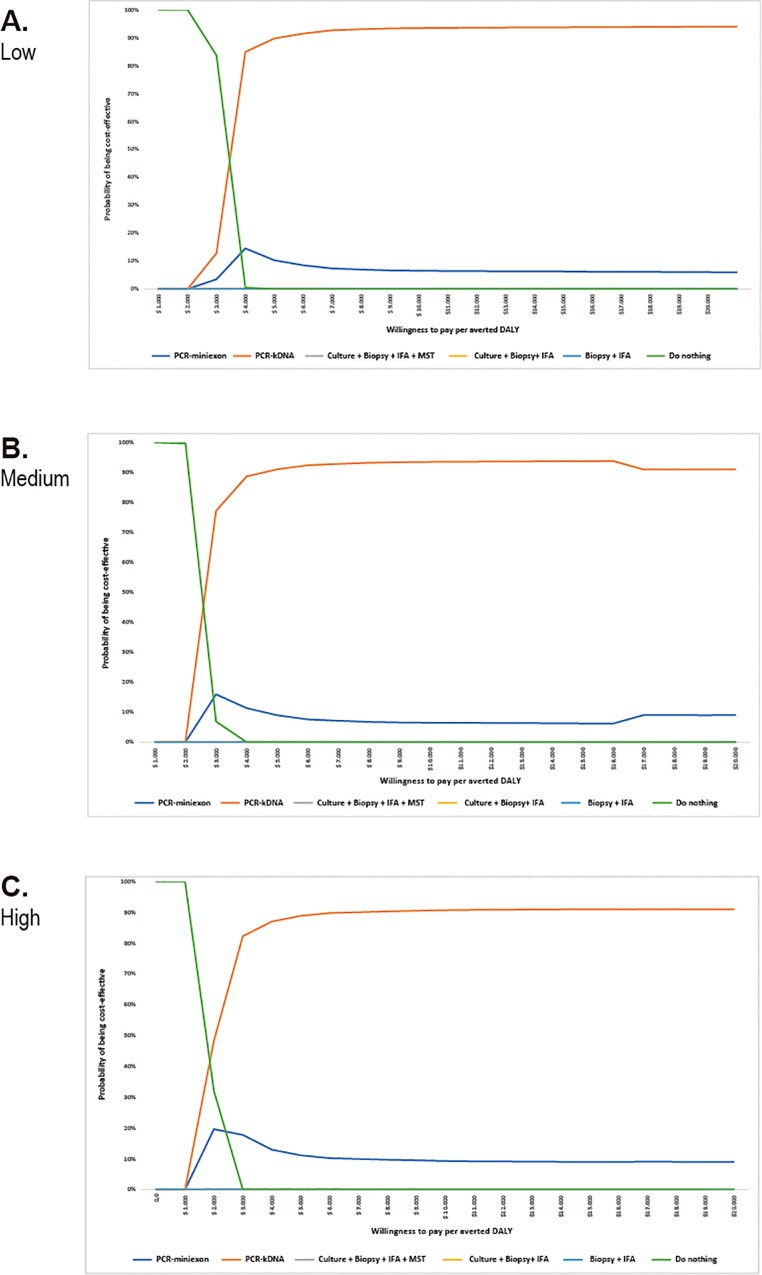
Acceptability curve of the alternatives analyzed for the levels of clinical suspicion, Colombia, 2015. (A) Low, (B) Medium, (C) High.

Acceptability curves show that the alternative PCR-kDNA is more likely to be the most cost-effective alternative for WTP greater than US$ 3560 per DALY averted at the level of low clinical suspicion. The WTP values are US$ 2820 and US$ 1930 per DALY averted at medium and high clinical suspicion levels, respectively. Alternatives of Culture + Biopsy + IFA + MST; Culture + Biopsy + IFA, and Biopsy + IFA are not cost-effective at any level of clinical suspicion (Fig 6).

## Discussion

ML diagnostic alternatives are cost-effective in Colombia at high, medium and low clinical suspicion settings, with a WTP threshold of three GDP*pc*. In all three scenarios, PCR-based tests (kDNA or miniexon) proved to be cost-effective choices, with a better cost-effectiveness profile for PCR-kDNA. In the case of low clinical suspicion, the alternatives, including combinations of culture, biopsy, IFA, and MST were strong dominated (costly and less effective).

There are diagnostic difficulties with ML considering the available tools that were evaluated in our analysis. For any evaluated alternative, there are important quantities of false positives and false negatives that generate health consequences due to poor diagnoses. False positives are related to the test’s specificity and involve treating a non-ML patient with a drug of high toxicity [39]. The first line drug, meglumine antimoniate (Glucantime^®^), in addition to the complexity of its administration, has side effects and serious reactions such as cardiac toxicity and hepatic, pancreatic and renal alterations [40], in addition to therapeutic failure and resistance, which are increasingly observed [41]. On the other hand, individuals classified as false negatives are associated with the test’s sensitivity because these patients do not receive a timely treatment, worsening the physical, psychological and social consequences, as well as situations that also affect the family and community [42].

Parameters of sensitivity and specificity of the diagnostic tests do not allow us to know directly the probability of correct diagnosis. For this purpose, we used the predictive values (positive and negative). In a population with high level of clinical suspicion, where the disease is frequent, we are more certain that a positive test results indicates the presence of disease and less certain that a negative result indicates absence of it. Other authors showed that predictive values observed are not universally applied [43]. Those results are consistent with our findings where the same diagnostic alternatives were more cost-effective at high level of clinical suspicion.

There are pros and cons of the use of PCR-based tests in endemic areas. Studies conducted in Brazil showed diagnostic advantages in the use of molecular tests as kDNA-PCR [44]; in Argentina they support this type of diagnosis [45] and recent analyzes already incorporate the use of real-time PCR [46]. However, some authors note that these tests are far from the usual clinical application in endemic areas due to costs [45,47]. They offer as alternative, the miniaturization of the PCR equipment, greater affordable, increasing the routine use in low income countries [46,48–50].

This study showed the benefits in the cost-effectiveness of molecular techniques compared to routine diagnostic methods and aims to contribute in decision making about the convenience of incorporating molecular biology methods for diagnosis, but to high costs. However, obtaining adequate samples in the realization of PCR techniques can reduce costs and improve the PCR’s cost-effectiveness profile. The use of cytology brushes (at a cost of US$ 0.20–0.50) is practical and comparatively affordable. It can be transported easily to a reference center for diagnostic testing and is likely appropriate for field situations, in addition to offering the advantage of differentiating mucosal lesions with simple non-invasive samples [9].

The main key driver of the cost-effectiveness of ML diagnostic tests is the treatment cost per case. An ML patient could be expensive for the health system to treat, but the costs are also very variable. It highlights the importance of a timely diagnosis because a delayed diagnosis is related to worse episodes. Larger or aggressive lesions, such as deformity or airway obstruction, are more difficult to treat and are related to higher health attention costs [39]. The availability of better diagnostic alternatives could have an impact and result in a larger reduction on health costs in Colombia, such as surgical procedures (reconstructive plastic surgery), which were not included in the present study but may be subject to further analysis. In addition, variables as cost of untreated case is not a key-driver parameter in the cost-effectiveness model for the best performed alternative (PCR kDNA) in spite of the importance in a clinical setting. It is because this alternative reduces significatively the proportion on nontreated cases.

Although it was not the most cost-effective alternative, the use of the PCR-miniexon test could have and additional advantage of allowing the identification of species of the genus Leishmania causing the disease. It could be included in the clinical practice guidelines to orient the treatment more accurately and result in a better cost-effectiveness profile of the PCR-miniexon alternative not evaluated in the present analysis. In countries such as Peru, it is important to identify species, where co-endemic problems have been reported, as well as different prognoses and responses to treatments [9]. On the other hand, the report of cases involving subspecies is increasing where the PCR-miniexon may be the best diagnostic alternative.

As far as we know, this is the first cost-effectiveness analysis of ML diagnosis in Colombia and in the international literature. Cost-effectiveness studies have been performed for the treatment of ML [51]; there is no evidence of research related to the diagnosis of this disease. Recently studies were been made a cost-effectiveness analysis in other types of leishmania as of diagnostic tests for human visceral leishmaniasis in Brazil [52] that highlights the benefits of PCR techniques as cost-effective public health measures and a study in Iran that compares three molecular methods for the diagnosis of cutaneous leishmaniasis [53]. Therefore, making an economic evaluation of the diagnostic tests in our country is of great relevance and provides evidence to decision makers when considering the inclusion of this type of evidence in clinical practice guidelines.

This analysis has limitations. First, the population with clinical suspicion has as a source the cases of mucosal leishmaniasis reported to the Sivigila, it is known that this data is underestimated, however it is the information of administrative bases available. Second, there is no information on the burden of ML disease in Colombia [54], so it was necessary to try to estimate it by simulating hypothetical cohorts of patients with different level of clinical suspicion. Third, a patient may have several opportunities to be diagnosed; however, to maintain the relative simplicity of the model, only one diagnostic opportunity for ML was considered. Fourth, clinical expert consensus asked for adjustments to the IFA sensitivity data based on their experience, which can be contrasted with the available literature [10]. Fifth, the costs were estimated in a reference center, so these could be different in other institutions, changing the cost-effectiveness values that were estimated. However, most of the Colombian ML cases are diagnosed and treated at the reference center. Sixth, follow up and treatment costs of adverse drug effects were included directly in the treatment costs per patient, but not as transition probability and additional model state. If the occurrence of adverse effects is different to our sample the estimated cost-effectiveness of the alternatives compared could be different. Seventh, ML frequently affects the nasal mucosa [3] but may compromise the nasal septum, palate, larynx and pharynx, causing facial disfiguration and difficulty in eating and speaking [55,56] with psychological repercussions [2,42], such as social isolation [23],which are situations that were not included in the present study but can be addressed in other studies.

## Conclusions

Diagnostic tests for ML based on PCR are the most cost-effective alternatives to a threshold of 3 GDP*pc* in patients with ML clinical suspicion, independent of their level of suspicion; thus, the use of these tests can be recommended for ML diagnosis through clinical practice guidelines. The PCR-kDNA alternative was the most cost-effective in the competitive scenario with the parameters included in the present model. Although was not evaluated here, the use of the PCR-miniexon could improve the identification of Leishmania species. In any case, other non-invasive methods for collecting samples suitable for the diagnosis of ML should be considered, which would reduce costs and discomfort to the patient and in turn improve diagnostic performance.

## Supporting information

S1 File Checklist. Consolidated Health Economic Evaluation Reporting Standards (CHEERS) list.
(DOC)

S2 File Supplementary tables of the model. (DOCX)

S3 File Model. Cost-effectiveness analysis of ML with PCR-based vs parasitological tests in Colombia.
(RAR)
