# Supplementary material for: Cost-effectiveness analysis of Mucosal Leishmaniasis diagnosis with PCR-based vs parasitological tests in Colombia
Source: PLoS One. 2019 Nov 4;14(11):e0224351. doi: 10.1371/journal.pone.0224351 (PMC6827906; doi:10.1371/journal.pone.0224351)
Supplement: S1 File — Consolidated Health Economic Evaluation Reporting Standards (CHEERS) list. (DOC) [file pone.0224351.s001.DOC]

**CHEERS list applied to the document Cost-effectiveness analysis of mucosal Leishmaniasis diagnosis with PCR-based vs parasitological tests in Colombia**

| **Section/item** | **Item No** | **Recommendation** | **Reported on page No/ line No** |
| --- | --- | --- | --- |
| **Title and abstract** | | | |
| Title | 1 | Identify the study as an economic evaluation or use more specific terms such as “cost-effectiveness analysis”, and describe the interventions compared. | page 1, line 4 |
| Abstract | 2 | Provide a structured summary of objectives, perspective, setting, methods (including study design and inputs), results (including base case and uncertainty analyses), and conclusions. | page 2, line 18 to 43 |
| **Introduction** | | | |
| Background and objectives | 3 | Provide an explicit statement of the broader context for the study. | page 3, line 46 to 60 |
|  |  | Present the study question and its relevance for health policy or practice decisions. | page 4, line 62 to 73 |
| **Methods** | | | |
| Target population and subgroups | 4 | Describe characteristics of the base case population and subgroups analysed, including why they were chosen. | Page 6, table 1;  page 9, line 109 to 113 |
| Setting and location | 5 | State relevant aspects of the system(s) in which the decision(s) need(s) to be made. | Page 5, line 83 to 88;  Page 5, line 99;  page 9, figure 1;  page 9, line119 to 124 |
|  |  |  |  |
| Study perspective | 6 | Describe the perspective of the study and relate this to the costs being evaluated. | page 9, line 125;  page 9, figure 1;  page 8 line 108 to 129 |
| Comparators | 7 | Describe the interventions or strategies being compared and state why they were chosen. | page 9, figure 1;  page 10 line 135 to 139;  page 10, figure 2;  page 10, figure 3; |
| Time horizon | 8 | State the time horizon(s) over which costs and consequences are being evaluated and say why appropriate. | page 11, line 153 to 157 |
| Discount rate | 9 | Report the choice of discount rate(s) used for costs and outcomes and say why appropriate. | page 8, tabla1;  page 10, line 128 to 129 |
| Choice of health outcomes | 10 | Describe what outcomes were used as the measure(s) of benefit in the evaluation and their relevance for the type of analysis performed. | page 11, line 157 to 162; |
| Measurement of effectiveness | 11a | *Single study-based estimates:*Describe fully the design features of the single effectiveness study and why the single study was a sufficient source of clinical effectiveness data. | Page 6, table 1  Effectiveness not is different among branches (our study is about diagnosis). Effectiveness was validated in the expert consensus |
|  | 11b | *Synthesis-based estimates*: Describe fully the methods used for identification of included studies and synthesis of clinical effectiveness data. |  |
| Measurement and valuation of preference based outcomes | 12 | If applicable, describe the population and methods used to elicit preferences for outcomes. | not applicable |
| Estimating resources and costs | 13a | *Single study-based economic evaluation:* Describe approaches used to estimate resource use associated with the alternative interventions. Describe primary or secondary research methods for valuing each resource item in terms of its unit cost. Describe any adjustments made to approximate to opportunity costs. | page 11, line 164 to 178;  page 12, line 180 to 197 |
|  | 13b | *Model-based economic evaluation:*Describe approaches and data sources used to estimate resource use associated with model health states. Describe primary or secondary research methods for valuing each resource item in terms of its unit cost. Describe any adjustments made to approximate to opportunity costs. | page 6, table 1  page 9, line 114 to 127  page 10, line 131 to 139 |
| Currency, price date, and conversion | 14 | Report the dates of the estimated resource quantities and unit costs. Describe methods for adjusting estimated unit costs to the year of reported costs if necessary. Describe methods for converting costs into a common currency base and the exchange rate. | page 9, line 114 to 129 |
| Choice of model | 15 | Describe and give reasons for the specific type of decision-analytical model used. Providing a figure to show model structure is strongly recommended. | page 10, line 131 to 134;  page 10, figure 3 |
| Assumptions | 16 | Describe all structural or other assumptions underpinning the decision-analytical model. | page 6-8, table 1 |
| Analytical methods | 17 | Describe all analytical methods supporting the evaluation. This could include methods for dealing with skewed, missing, or censored data; extrapolation methods; methods for pooling data; approaches to validate or make adjustments (such as half cycle corrections) to a model; and methods for handling population heterogeneity and uncertainty. | page 9, line 108 to 210; |
| **Results** | | | |
| Study parameters | 18 | Report the values, ranges, references, and, if used, probability distributions for all parameters. Report reasons or sources for distributions used to represent uncertainty where appropriate. Providing a table to show the input values is strongly recommended. | page 6, line 101, table 1; |
| Incremental costs and outcomes | 19 | For each intervention, report mean values for the main categories of estimated costs and outcomes of interest, as well as mean differences between the comparator groups. If applicable, report incremental cost-effectiveness ratios. | page 15, line 237, table 3;  page 16, line 250, table 4;  page 17, line 257, table 5; |
|  |  |  |  |
| Characterising uncertainty | 20a | *Single study-based economic evaluation:* Describe the effects of sampling uncertainty for the estimated incremental cost and incremental effectiveness parameters, together with the impact of methodological assumptions (such as discount rate, study perspective). | not applicable |
|  | 20b | *Model-based economic evaluation:*Describe the effects on the results of uncertainty for all input parameters, and uncertainty related to the structure of the model and assumptions. | page 18, line 274 to 279;  page 18, figure 5;  page 19, line 284 to 290;  page 19, figure 6 |
| Characterising heterogeneity | 21 | If applicable, report differences in costs, outcomes, or cost-effectiveness that can be explained by variations between subgroups of patients with different baseline characteristics or other observed variability in effects that are not reducible by more information. | not applicable |
| **Discussion** | | | |
| Study findings, limitations, generalisability, and current knowledge | 22 | Summarise key study findings and describe how they support the conclusions reached. Discuss limitations and the generalisability of the findings and how the findings fit with current knowledge. | page 19, line 295 to  page 23, line 375 |
| **Other** | | | |
| Source of funding | 23 | Describe how the study was funded and the role of the funder in the identification, design, conduct, and reporting of the analysis. Describe other non-monetary sources of support. | Information provided via the submission system  page 23, line 391 to  page 24, line 395 |
| Conflicts of interest | 24 | Describe any potential for conflict of interest of study contributors in accordance with journal policy. In the absence of a journal policy, we recommend authors comply with International Committee of Medical Journal Editors recommendations. | Information provided via the submission system  page 23, line 389 |

For consistency, the CHEERS statement checklist format is based on the format of the CONSORT statement checklist
