# Supplementary material for: Cost-effectiveness analysis of Mucosal Leishmaniasis diagnosis with PCR-based vs parasitological tests in Colombia
Source: PLoS One. 2019 Nov 4;14(11):e0224351. doi: 10.1371/journal.pone.0224351 (PMC6827906; doi:10.1371/journal.pone.0224351)
Supplement: S2 File — (DOCX) [file pone.0224351.s002.docx]

**Table 1. Incidence of LM according Colombia surveillance system.**

| **Year** | **Cases** | **Population** | **Rate** |
| --- | --- | --- | --- |
| 2007 | 82 | 43926929 | 0.00000187 |
| 2008 | 89 | 44451147 | 0.00000200 |
| 2009 | 108 | 44978832 | 0.00000240 |
| 2010 | 163 | 45509584 | 0.00000358 |
| 2011 | 165 | 46044601 | 0.00000358 |
| 2012 | 202 | 46581823 | 0.00000434 |
| 2013 | 141 | 47121089 | 0.00000299 |
| Total | 950 | 318,614,005 | 0.00000298 |

**Table 2. Other causes mortality (excluding Leishmaniasis), according the National Statistics Department (DANE).**

| **Age in years** | **Deaths** | **Population** | **Rate** |
| --- | --- | --- | --- |
|  | **A** | **B** | **A/B** |
| U1P | 181074 | 14802930 | 0.01223 |
| 1-4 | 39416 | 56187951 | 0.00070 |
| 5-9 | 19915 | 70931218 | 0.00028 |
| 10-14 | 23420 | 70297567 | 0.00033 |
| 15-19 | 82623 | 66646476 | 0.00124 |
| 20-24 | 121432 | 60693149 | 0.00200 |
| 25-29 | 115727 | 54461200 | 0.00212 |
| 30-34 | 104058 | 50244567 | 0.00207 |
| 35-39 | 100240 | 46982500 | 0.00213 |
| 40-44 | 103785 | 43407750 | 0.00239 |
| 45-49 | 113995 | 37914582 | 0.00301 |
| 50-54 | 131143 | 31124406 | 0.00421 |
| 55-59 | 151139 | 24632305 | 0.00614 |
| 60-64 | 186725 | 19479604 | 0.00959 |
| 65-69 | 230964 | 15279168 | 0.01512 |
| 70-74 | 287231 | 11764971 | 0.02441 |
| 75-79 | 317686 | 8895508 | 0.03571 |
| 80+ | 741191 | 8088743 | 0.09163 |
| Total | 3051764 | 691834595 | 0.00441 |

U1P: Under one year population

**Table 3. Mortality due to leishmania in Colombia.**

| **Age in years** | **Deaths** | **Population** | **Rate** |
| --- | --- | --- | --- |
|  | **A** | **B** | **A/B** |
| U1P | 0 | 13883222 | 0.0 |
| 1-abr | 0 | 56187951 | 0.0 |
| 5-sep | 0 | 70931218 | 0.0 |
| oct-14 | 0 | 70297567 | 0.0 |
| 15-19 | 0 | 66646476 | 0.0 |
| 20-24 | 0 | 60693149 | 0.0 |
| 25-29 | 1 | 54461200 | 0.000000018 |
| 30-34 | 1 | 50244567 | 0.000000020 |
| 35-39 | 1 | 46982500 | 0.000000021 |
| 40-44 | 0 | 43407750 | 0.000000000 |
| 45-49 | 1 | 37914582 | 0.000000026 |
| 50-54 | 1 | 31124406 | 0.000000032 |
| 55-59 | 0 | 24632305 | 0.000000000 |
| 60-64 | 1 | 19479604 | 0.000000051 |
| 65-69 | 2 | 15279168 | 0.000000131 |
| 70-74 | 1 | 11764971 | 0.000000085 |
| 75-79 | 0 | 8459804 | 0.0 |
| 80+ | 2 | 8088743 | 0.000000247 |
| **Total** | 11 | 690479183 | 0.000000016 |

**Table 4. Report of average use per patient and cost item.**

| **Items** | **Total** | **Average per patient** | **Total Cost (US$)** |
| --- | --- | --- | --- |
| **Consultations** |  |  |  |
| Dermatology | 357 | 7.4375 | 4,477.42 |
| Follow up Consultation | 204 | 4.2500 | 2,558.53 |
| Surgical valuation | 35 | 0.7292 | 438.96 |
| Social-work valuation | 10 | 0.2083 | 42.23 |
| Otorhinolaryngology | 9 | 0.1875 | 106.30 |
| Internal medicine | 6 | 0.1250 | 75.25 |
| Urgency consultation | 3 | 0.0625 | 17.43 |
| Cardiology | 2 | 0.0417 | 25.08 |
| **Procedures** |  |  |  |
| Nose biopsy | 55 | 1.1458 |  |
| Direct smear | 9 | 0.1875 | 40.01 |
| Skin biopsy | 6 | 0.1250 |  |
| Palate biopsy | 1 | 0.0208 |  |
| **Lab tests** |  |  |  |
| Indirect immunofluorescence assay (IFA) | 126 | 2.6250 | 3,120.53 |
| Routine stains | 55 | 1.1458 | - |
| Alanine aminotransferase | 39 | 0.8125 | 216.70 |
| Aspartate aminotransferase | 39 | 0.8125 | 216.70 |
| Amilasa | 38 | 0.7917 | 211.15 |
| Creatinine | 28 | 0.5833 | 97.79 |
| Montenegro skin test (MST) | 23 | 0.4792 | - |
| Electrocardiogram | 22 | 0.4583 | 97.76 |
| Culture-leishmania | 20 | 0.4167 | - |
| Hemogram | 17 | 0.3542 | 105.26 |
| Other (Partial urine, Thyroid stimulating hormone, Beta human chorionic gonodotropin, X-ray of paranasal sinuses, Immunodiffusion histoplasmosis) | 11 | 0.2292 | 77.76 |
| Alkaline Fosfatasa | 7 | 0.1458 | 27.78 |
| Blood Urea Nitrogen (BUN) | 6 | 0.1250 | 16.38 |
| Antiodies to Human Immunodeficiency Virus (HIV) | 5 | 0.1042 | 81.76 |
| Bilirubin-direct | 4 | 0.0833 | 15.24 |
| Venereal disease research laboratory (VDRL) | 4 | 0.0833 | 9.53 |
| Polymerase chain reaction (PCR) | 3 | 0.0625 | 141.52 |
| Review of sheets | 3 | 0.0625 | 46.67 |
| Bilirubin-total | 2 | 0.0417 | 7.62 |
| Glucose | 2 | 0.0417 | 6.35 |
| Bilirubin-indirect | 1 | 0.0208 | - |
| Culture-hongos | 1 | 0.0208 | 27.62 |
| Culture-mycobacteria | 1 | 0.0208 | 16.35 |
| PCR miniexon | 1 | 0.0208 | 47.17 |
| Cutaneous tuberculosis test PPD | 1 | 0.0208 | - |
| Pregnancy test | 1 | 0.0208 | 5.72 |
| X-ray chest | 1 | 0.0208 | 15.65 |
| Paranasales CT scan | 1 | 0.0208 | 119.77 |
| **Medicines** |  |  |  |
| Meglumine antimoniate (Glucantime®) (vial 405gr / 5cc) | 4769 | 99.3542 | 3,719.20 |
| Miltefosine (tablet 50 mg) | 84 | 1.7500 | 28.82 |
| Sodium stibogluconate (Pentostam®) vial 1.56 ml | 42 | 0.8750 | 71.05 |
